# Phonological encoding in Tongan: An experimental investigation

**DOI:** 10.1177/17470218221138770

**Published:** 2022-12-01

**Authors:** Katsuo Tamaoka, Jingyi Zhang, Masatoshi Koizumi, Rinus G Verdonschot

**Affiliations:** 1 School of Foreign Languages, Hunan University, Changsha, China; 2 Graduate School of Humanities, Nagoya University, Nagoya, Japan; 3 Center for Language and Cultural Studies, University of Miyazaki, Miyazaki, Japan; 4 Graduate School of Arts and Letters, Tohoku University, Sendai, Japan; 5 Max Planck Institute for Psycholinguistics, Nijmegen, The Netherlands

**Keywords:** Language production, Tongan, picture–word naming, prosodification, phonological encoding

## Abstract

This study is the first to report chronometric evidence on Tongan language production. It has been speculated that the mora plays an important role during Tongan phonological encoding. A mora follows the (C)V form, so /a/ and /ka/ (but not /k/) denote a mora in Tongan. Using a picture–word naming paradigm, Tongan native speakers named pictures containing superimposed non-word distractors. This task has been used before in Japanese, Korean, and Vietnamese to investigate the initially selected unit during phonological encoding (IPU). Compared with control distractors, both onset and mora overlapping distractors resulted in faster naming latencies. Several alternative explanations for the pattern of results—proficiency in English, knowledge of Latin script, and downstream effects—are discussed. However, we conclude that Tongan phonological encoding likely natively uses the phoneme, and not the mora, as the IPU.

Speaking is a complex ability, yet it is carried out quickly and with few errors (Levelt, 1989). Most theoretical models agree that the production of speech can be divided between accessing the meaning of a word on one hand and the construction of its pronunciation on the other (e.g., Caramazza, 1997; Dell, 1986; Levelt et al., 1999; Roelofs, 2015). In the formation of the pronunciation of a word (e.g., “cymbal”) it has been suggested that three pieces of information are accessed (Levelt et al., 1999). Specifically (1) its morphemic make-up (e.g., < cymbal > is a free-standing singular morpheme), which in turn activates (2) its metrical information (e.g., ω = σ’σ meaning that the word [ω] is bi-syllabic [σσ] with stress [‘] on the first syllable), and (3) its constituent segments (e.g., /s/ /ɪ/ /m/ /b/ /ə/ /l/). The segments are then combined with the metrical frame to form syllabified representations [‘sɪm] [bəl]. One reason for such an elaborate process involves the phenomenon of re-syllabification. This phenomenon occurs when one segment is assigned to a different adjoining syllable. For example, in the English sentence “I said it” ([aɪ] [se] [dit]) the /d/ in “said” [sed] would be assigned to the last syllable [it] to form [dit]. However, in languages such as Mandarin Chinese, which does not employ re-syllabification, this phenomenon does not occur, hence the assignment of phonological information into a metrical structure might be dissimilar.

There has been an upsurge in the literature indicating that the “units” and “frames” used in constructing a phonological word are indeed different among languages. For example, O’Séaghdha et al. (2010), You et al. (2012), Wang et al. (2018), and Zhang and Damian (2019) all showed that *syllables* are the *initial phonological unit* (hereafter, IPU) selected during Mandarin Chinese phonological encoding (but see Qu et al., 2020; Verdonschot et al., 2015). Similarly, Kureta et al. (2006), Verdonschot et al. (2011), and Ida et al. (2015) showed that in Japanese, the *mora* rather than the phoneme is the IPU. For Cantonese it has been proposed that both syllabic and sub-syllabic units (e.g., the rhyme: Wong & Chen, 2008, 2009; and the word-initial body: Wong et al., 2012), but never the onset by itself, can be activated in the early stages of phonological encoding. Recent studies, however, using both behavioural (Wong et al., 2018) and event-related potential (ERP) measures (Wong et al., 2019) have shown that effects related to syllable priming occur earlier than those of body-related priming indicating that the first selected phonological unit in Cantonese might be the syllable.

For Korean, the picture is less clear with mixed findings including evidence for the phoneme (e.g., Han & Verdonschot, 2019; Witzel et al., 2013), the syllable body (Li et al., 2021), and the full syllable as the IPU (Verdonschot et al., 2021). The notion that the IPU might be different for various languages has also found support in neurocorrelational electroencephalogram (EEG) studies (e.g., Mandarin: Qu et al., 2020; Wang et al., 2017; Japanese: Cantonese: Wong et al., 2019; Verdonschot et al., 2019) showing task-correlated brain activation only for particular IPUs for these respective languages. See Alderete and O’Séaghdha (2022) for a recent overview and cross-linguistic perspective on phonological encoding.

One interesting related tangent is the question of how bilinguals initially construct phonology when their two languages differ in their IPUs. Here, it has been found that proficiency level may strongly matter. For example, Nakayama et al. (2016) showed in an L2 speech production task that low-proficiency Japanese–English bilinguals rely heavily on their Japanese L1 (mora) IPU when speaking in their L2 while highly proficient bilinguals develop a more “native-like” phonemic IPU in their L2. Thus, they suggested that extensive exposure to L2 phonology could be important in developing a native-like IPU.

One consequence of having a non-native IPU as in the case of low-proficiency Japanese-English bilinguals is that L2-pronunciation may be affected. For example, Verdonschot and Masuda (2020) showed that when analysing the acoustic wave forms of low- and high-proficiency Japanese-English bilinguals significantly more cases of epenthesis occurred for the low-proficiency group (e.g., “scar” would have been pronounced as “sukaa” or “sukaru”) resulting in the L2 conforming to the IPU of L1.

The current article is concerned with phonological encoding during speech production in Tongan, a Polynesian language spoken in the South Pacific Kingdom of Tonga. The island nation of Tonga recently attracted global media attention due to the January 2022 eruption of a submarine volcano and its resulting tsunami.

Tongan phonological encoding is of interest as there are indications that, as in Japanese, the mora may be the initial phonological unit selected during production. Hayes (1995) considers the basic rhythmic unit in Tongan to be a combination of moras with a strong first and a weak second mora. The Tongan language has a rather limited inventory of twelve consonants (p, m, f, v, t, s, n, k, l, ŋ, ʔ, and h) and five vowels (i, e, a, u, o; cf. Garellek & White, 2015). It has a rigid syllabic structure and permits only (C)V syllables (Anderson & Otsuka, 2006) and therefore cannot manifest re-syllabification. Mono-moraic words in Tongan typically serve a grammatical function (e.g., “ki” meaning “to/towards,” “pe” meaning “or”) as opposed to words carrying meaning (e.g., “kolo” meaning “town”) which have more than one mora. A simple rule to determine the number of moras in a Tongan word is to simply “count the vowels.” For example, “feke” meaning “octopus” has two vowels, therefore two moras (/fe/+/ke/). Another example is “fua” (/fu/+/a/) meaning “fruits.” A phrase such as “mālō e lelei” (meaning “hello” in Tongan) is transcribed as /ma/+/a/+/lo/+/o/+/e/+/le/+/le/+/i/ and has eight moras (note: the macron ^—^ in mā and lō indicates a long vowel, and has a duration of two moras). According to Taumoefolau (2002) and Anderson and Otsuka (2006), it is also possible to determine the number of *syllables* by simply counting the vowels, therefore the number of moras and syllables are suggested to be coextensive in Tongan (Anderson & Otsuka, 2006, p. 41). Tongan is a stress language and the primary stress almost always falls on the penultimate mora of a word (Garellek & Tabain, 2020). For example, the word mā (“/ma/+/a/) ‘bread” is stressed on the first mora (/ma/) although there are exceptions such as when a word is followed by a clitic (e.g., “ni” meaning “that”) as in “that bread” – “mā ni” (/ma/+‘/a/+/ni/) which would have stress on the second mora /a/.

English loanwords in Tongan often show epenthesis or consonant deletion to adhere to Tongan phonotactics (like Japanese; see Verdonschot & Masuda, 2020). For instance, Schutz (1970) mentions several examples such as: “plastic” → “palasitiki” and “horse” → “hoosi.” Zuraw et al. (2019) mention similar examples such as “kangaroo” → /kaŋikaluu/ and “monogram” → /monokalame/.

In all, it seems that Tongan has several important traits in common with Japanese including its phonological encoding system which ostensibly uses the mora (or syllable) as the initial unit to create a word (Roelofs, 2015; Verdonschot et al., 2011). However, several other factors might be of relevance here; the first one being that both Tongan and English are official languages of the Kingdom. This situation has arisen from the fact that, although it was never formally colonised, Tonga was a former British protectorate from 1900 until 1970 after which time it became an independent nation within the British Commonwealth. Consequently, most Tongan speakers have enjoyed a comparatively high-level of education and are therefore relatively fluent in English as well. This is important as it has been argued for Chinese-English bilinguals that increased proficiency in an L2 (English) may lead to sub-syllabic priming effects even in their L1 (Chinese: Verdonschot et al., 2013). In other words, the answer to whether Tongan is moraic or not, may potentially be obscured by their high L2 (English) proficiency. A second important factor is that Tongan is written using the Latin script (unlike Japanese which is written using a combination of moraic and logographic scripts). Consequently, Tongan orthography denotes individual phonemes which may perhaps have instilled phonemic awareness into native speakers.

To investigate whether the phoneme or the mora is the first unit selected during phonological encoding in Tongan we conducted a picture–word naming experiment in which participants named pictures onto which non-word distractors were superimposed. Most language production models agree that there are connections between the perception and production networks for speech and that visual word recognition also involves phonological activation. Levelt et al. (1999) further assume that active phonological units in the perceptual network can directly affect the corresponding IPUs in the production lexicon (Levelt et al. 1999, p. 7 “assumption 2”). Therefore, we have chosen non-words as distractors as they are proposed to directly activate IPUs in the production network while reducing lexical influences. Employing PWIs with non-word distractors has been carried out several times before (see Verdonschot et al., 2019 for a similar experiments in Japanese; Verdonschot et al., 2021 for Vietnamese; and Verdonschot et al., 2022 for Korean) although it should be noted that task might be susceptible to orthographic confounds

In our experiment the non-word distractors were either onset- or mora-related. If the initial phonological unit selected in Tongan is the mora we expect facilitation only for the mora-overlap condition and not for the onset-overlap condition. If, however, the initial phonological unit selected in Tongan is the phoneme, then facilitation is expected for both conditions. As far as we know, there is currently no chronometric data on Tongan language production available, therefore this article informs theories of language production through insights from rarely studied languages, such as Tongan, providing a broader cross-language perspective (see also Alderete & O’Séaghdha, 2022 who similarly argue to move beyond Indo-European languages).

## Experiment—picture–word naming using Tongan non-word distractors

### Method

#### Participants

Twenty-four native Tongan speakers from the Tonga Institute of Education in Nuku’alofa, Tonga (14 female; age 22.3 ± 5 years) took part in this experiment and were compensated for their participation. All spoke English as their L2. Participants self-rated four aspects of their English ability on a 1 (no-proficiency) to 6 (native-proficiency) scale as speaking (4.4 ± 0.9), listening (4.6 ± 1.1), reading (4.8 ± 1), writing (4.6 ± 1.1) and also marked their daily Tongan/English use as a percentage (average: 77 ± 12% Tongan vs. 23 ± 12% English). Informed consent was obtained from all participants and the experiment was carried out in accordance with the Declaration of Helsinki.

#### Materials and design

Forty picturable bi-moraic words were selected (see online Supplementary Material), each accompanied by four non-word distractors and their respective controls, totaling 160 stimuli per participant. For example, the Tongan word “feke” meaning “octopus” had the onset /f/ overlapping with the non-word distractor “folu” (with non-word control “solu”), and the mora /fe/ with the non-word distractor “felu” (with non-word control “selu”). All stimuli were created by a native Tongan speaker. We opted to use non-words as distractors as there are currently no lexical characteristics databases available for Tongan and non-words have been shown to be effective in eliciting responses (e.g., Verdonschot et al., 2019, 2021, 2022).

#### Apparatus and procedure

Participants were seated in a quiet room in front of a computer screen. E-Prime 3.0 was used to present stimulus materials and record naming latencies (RTs) and errors (Spapé et al., 2019). Participants first saw a fixation cross for 1,000 ms followed by the picture which they were asked to name. The picture was removed once a response was given. If there was no response within 2,500 ms, the picture disappeared from the screen. For each trial the experimenter (native Tongan speaker) judged whether the response was accurate, contained an error (e.g., saying cat instead of dog), or contained a voice key error (e.g., non-speech sounds triggering the voice key). Between each trial there was a blank 500-ms interval. Participants were asked to name targets in four blocks. Target words were shown only once per block. Block order was counterbalanced using a Latin-square design. For each participant individually randomised lists were created per block such that successive targets were not be semantically or phonologically related. For example, targets such as *owl* “veka” could not directly precede or follow targets such as *dove* “lupe”; also, targets such as *kite* “lofa” or *window* “luva” could not directly precede or follow targets such as *dove* “lupe.”

#### Results

About 2.6% of the RTs were discarded due to (1) a failure to respond, (2) stuttering or correcting a response, (3) triggering the voice key using a non-verbal response (e.g., coughing), or (4) a failure to trigger the voice key. Furthermore, there were 1.4% errors (e.g., wrong words). The treatment of correct RT data for this analysis was as follows: First, trials which exceeded 2.5 *SD* (3.2%) were excluded. The remaining 3,564 data points were used for further analysis (see Table 1 for RTs and accuracy information). A comparison of raw RTs, log-transformed RTs, and inverse-transformed RTs (i.e., –1,000/RT) revealed that inverse-transformed RTs were closest to normality and were therefore used in subsequent analyses. Error data was not further analysed as there were few errors which were equally distributed among conditions. Response latencies were analysed with a linear mixed effects model with participants and items as crossed random effects (e.g., Baayen, 2008) using the “lme4” package (Bates et al., 2015) implemented in R 4.0.3 (R Core Team, 2021). The “lmerTest” package in R was used to calculate the *p*-values using Satterthwaite’s approximation for the degrees of freedom Kuznetsova et al., 2017). In the experiment we opted to use an incremental modelling approach (Matuschek et al., 2017) to establish the most optimal statistical model for our data.

**Table 1.** RTs and standard deviation (*SD*) in ms.

Target “feke”	C-Distractor RT (*SD*)—example	%E	CV-Distractor RT (*SD*)—example	%E
Related	780 (220)—“folu”	.5	749 (200)—“felu”	.2
Control	800 (228)—“solu”	.4	777 (210)—“selu”	.3
Effect	20 ms		28 ms	

%E = percentage errors (within condition); RT = reaction time, C = consonant, V = vowel.

We considered the following variables: “Trial” (centred) denoting how far a participant had progressed in the experiment, “Congruency” with two levels (i.e., overlap, control) and “OverlapSize” with two levels (i.e., Onset, CV). The factor Congruency was deviation contrast-coded (–.5, .5). The final model using the incremental modelling approach was invRT ~ Trial_centered_ + Congruency + (1|Participant) + (1|Item). See Table 2 for more details. Although approaching significance, there was no significant main effect of OverlapSize (*t* = –1.831; *p* = .06). The model including OverlapSize was also not significantly better (χ^2^ = 3.36) than the model without. The interaction between Congruency (overlap or not) and OverlapSize (C and CV) was far from significant (*t* = –0.346; *p* = .73), and a model including the interaction also did not provide a better fit to the data (χ^2^ = 0.12) and was therefore not included.

**Table 2.** Experimental results.

Fixed Effects	Estimate	*SE*	*df*	*t*-value	Pr > (|*t*|)
(intercept)	–1.37	0.03	28.8	–48.785	0.001
Trial_centred_	–0.07	0.01	3476.0	–14.824	0.001
Congruency	–0.05	0.02	156.8	–2.322	0.023

SE: standard error.

#### Discussion

This article set out to investigate whether native Tongan speakers, a language which displays moraic qualities has an initially selected mora-based phonological unit (like Japanese) or a phoneme-based (like English). When participants named pictures it was found that both onset overlapping distractors, and mora overlapping distractors facilitated naming latencies. Therefore, it seems most likely that the phoneme is the initially selected unit during Tongan phonological encoding.

However, there are some alternative explanations for the appearance of onset effects. First, participants might have been sufficiently proficient in L2-English for an onset effect to appear in their L1 (see Verdonschot et al., 2013 for a similar reasoning with highly-proficiency Chinese-English bilinguals). To further investigate this, we used participants’ English self-ratings and English usage scores as variables in the model. However, no significant effects of these variables appeared (all *t*s <= 1).

Second, it may have been that the use of the Latin script has made Tongan native speakers aware of phonemes in the language. For example, for Japanese, Inagaki, Hatano & Otake (2000) showed that pre-literate Japanese children used a syllabic unit during word segmentation prior to learning hiragana (a moraic script). Children were shown pictures and a series of empty circles while holding a doll in their hands. Their task was to say the name of the picture and “jump the doll” in the circles at the same time matching the name of the picture. So, the number of circles they jumped indicated how the child segmentated the word during production. For example, saying /kani/ (crab) caused two jumps (both for literate/illiterate children). However, saying paNda (three mora, /pa/ /N/ /da/ but two syllables /paN/ /da/) caused two jumps for illiterate children which is the number of syllables but three jumps for literate children, which is the number of mora, as clearly represented by the script パンダ). Whether or not script indeed instils phonemic awareness was not specifically assessed in the current experiment. However, some the language games played by pre-literate Tongan children, such as “matetupu’a,” a riddle game in which a “thing” is described, for example: “I am a fruit” and “I hang on a tree,” and “I start with the letter S” does *anecdotally* inform us that Tongan children are at least aware of individual phonemes in their language even if they could not yet read. Naturally, games may involve manipulations that are not part of typical encoding procedures therefore they do not provide strong, and only anecdotal, information.

Third, one could postulate that our onset effect in fact takes place downstream as syllables can be decomposed further into segmental units in later speech production stages.^1^ In other words, our onset effect is not a genuine IPU effect, though the CV effect is. Although this cannot be ruled out, it seems puzzling then why other languages such as Japanese, Cantonese, and Mandarin which also have downstream processes (e.g., O’Séaghdha et al., 2010) have been investigated with similar (PWI) tasks and no onset effects have been reported for these languages.

In all, it might simply be the case that Tongan, despite any presumed moraic qualities, uses the phoneme as the initially selected unit during language production. Tongan speech errors, for example, do involve single phoneme manipulations (e.g., one might erroneously say: “leke fahi*” instead of “feke lahi” “big octopus”). This contrasts with Japanese which mostly displays moraic and not phonemic speech errors (e.g., Kubozono, 1989). Note that the existence of phoneme errors in a language in itself should be taken with caution. In Dutch, for example, there are indeed many phoneme errors when compared with syllabic errors. However, Alderete (2022) has shown that phoneme slips are also relatively frequent in Chinese (e.g., additions such as /uk55/ → luk55 “house”), yet, significant numbers of syllable errors also exist in Cantonese^1^. However, the existence of phoneme slips in Cantonese does not mean that the Cantonese IPU is the phoneme (as far as we know no significant onset effects are found in Cantonese experiments investigating the IPU). As no database on speech errors for Tongan exists it remains unclear what the precise distribution between phoneme and moraic errors in Tongan is and a speech error corpus needs to be created to allow for a more comprehensive view on what speech errors can tell us about Tongan language production.

For now, our data do seem to point towards the phoneme as the initially selected unit during Tongan phonological encoding, irrespective of L2 proficiency or script. However, to investigate alternative explanations such as the ones mentioned earlier, replication and extension of our findings preferentially with additional paradigms (e.g., form priming) is warranted.

## Supplemental Material

sj-docx-1-qjp-10.1177_17470218221138770 – Supplemental material for Phonological encoding in Tongan: An experimental investigation Click here for additional data file. Supplemental material, sj-docx-1-qjp-10.1177_17470218221138770 for Phonological encoding in Tongan: An experimental investigation by Katsuo Tamaoka, Jingyi Zhang, Masatoshi Koizumi and Rinus G Verdonschot in Quarterly Journal of Experimental Psychology

## Notes

1 We would like to thank an anonymous reviewer for pointing this out.
