# Supplementary material for: Phonological encoding in Tongan: An experimental investigation
Source: Q J Exp Psychol (Hove). 2022 Dec 1;76(10):2226–31. doi: 10.1177/17470218221138770 (PMC10503232; doi:10.1177/17470218221138770)
Supplement: sj-docx-1-qjp-10.1177_17470218221138770 – Supplemental material for Phonological encoding in Tongan: An experimental investigation [file sj-docx-1-qjp-10.1177_17470218221138770.docx]

Supplementary Material for:

1. **Phonological encoding in Tongan: an experimental investigation**
2. Katsuo Tamaoka
3. School of Foreign Languages, Hunan University, Changsha, China
4. Graduate School of Humanities, Nagoya University, Nagoya, Japan

Jingyi Zhang

Center for Language and Cultural Studies, University of Miyazaki, Miyazaki, Japan

Masatoshi Koizumi

Graduate School of Arts and Letters, Tohoku University, Sendai, Japan

1. Rinus G. Verdonschot
2. Max Planck Institute for Psycholinguistics, Nijmegen, The Netherlands

Stimuli used in the experiment. The distractors were all non-words.

| Target Picture | Translation | C-overlap | C-control | CV-overlap | CV-control |
| --- | --- | --- | --- | --- | --- |
| feke | octopus | folu | solu | felu | selu |
| fonu | turtle | fami | tami | fomi | tomi |
| kuli | dog | kelu | nelu | kulu | nulu |
| namu | mosquito | neimo | seimo | naemo | saemo |
| paka | crab | pivo | tivo | pavo | tavo |
| toke | eel | tise | mise | tose | mose |
| veka | owl | vinu | tinu | venu | tenu |
| lupe | dove | loni | poni | luni | puni |
| mafu | heart | mueha | lueha | maeha | laeha |
| mata | eyes | mepe | fepe | mape | fape |
| nifo | teeth | nanu | kanu | ninu | kinu |
| tuhu | finger | tota | hota | tuta | huta |
| lole | candy | lufi | fufi | lofi | fofi |
| moli | orange | maiti | faiti | mouti | fouti |
| pani | bun | pohe | hohe | pahe | hahe |
| fale | house | foupu | koupu | faopu | kaopu |
| fuka | flag | falo | salo | fulo | sulo |
| hele | knife | huoki | tuoki | heiki | teiki |
| helu | comb | hunge | nunge | henge | nenge |
| veve | garbage | viase | liase | veose | leose |
| kane | bucket | kaoni | ngaoni | kauni | ngauni |
| kulo | pot | kafe | nafe | kufe | nufe |
| lofa | kite | lualo | pualo | loalo | poalo |
| luva | window | lesa | tesa | lusa | tusa |
| maka | battery | mofa | fofa | mafa | fafa |
| nafa | drum | nilu | vilu | nalu | valu |
| puha | box | puome | muome | pueme | mueme |
| pula | balloon | pasa | lasa | pusa | lusa |
| sila | envelope | sehi | kehi | sihi | kihi |
| sote | shirt | siahu | piahu | soihu | poihu |
| tele | razor | tuko | suko | teko | seko |
| tohi | book | teisu | heisu | tousu | housu |
| vaka | boat | vome | nome | vame | name |
| kato | bag | kima | mima | kama | mama |
| kofu | dress | kufa | fufa | kofa | fofa |
| leta | belt | laehi | kaehi | leihi | keihi |
| piva | skirt | pasu | masu | pisu | misu |
| vesa | bracelet | voafo | loafo | veafo | leafo |
| motu | island | mine | ngine | mone | ngone |
| pona | knot | peli | leli | poli | loli |
